# User-Accessible Machine Learning Approaches for Cell Segmentation and Analysis in Tissue

**DOI:** 10.3389/fphys.2022.833333

**Published:** 2022-03-10

**Authors:** Seth Winfree

**Affiliations:** Department of Pathology and Microbiology, University of Nebraska Medical Center, Omaha, NE, United States

**Keywords:** machine learning, deep learning—artificial neural network, segmentation, classification, neighborhoods, microenviroment, bio-imaging tools

## Abstract

Advanced image analysis with machine and deep learning has improved cell segmentation and classification for novel insights into biological mechanisms. These approaches have been used for the analysis of cells *in situ*, within tissue, and confirmed existing and uncovered new models of cellular microenvironments in human disease. This has been achieved by the development of both imaging modality specific and multimodal solutions for cellular segmentation, thus addressing the fundamental requirement for high quality and reproducible cell segmentation in images from immunofluorescence, immunohistochemistry and histological stains. The expansive landscape of cell types-from a variety of species, organs and cellular states-has required a concerted effort to build libraries of annotated cells for training data and novel solutions for leveraging annotations across imaging modalities and in some cases led to questioning the requirement for single cell demarcation all together. Unfortunately, bleeding-edge approaches are often confined to a few experts with the necessary domain knowledge. However, freely available, and open-source tools and libraries of trained machine learning models have been made accessible to researchers in the biomedical sciences as software pipelines, plugins for open-source and free desktop and web-based software solutions. The future holds exciting possibilities with expanding machine learning models for segmentation via the brute-force addition of new training data or the implementation of novel network architectures, the use of machine and deep learning in cell and neighborhood classification for uncovering cellular microenvironments, and the development of new strategies for the use of machine and deep learning in biomedical research.

## Introduction

Image use in the biomedical sciences varies from demonstrative and representative to data for quantitative interrogation. Quantitative analyses of tissue and cells, the basic building blocks in biology, requires the accurate segmentation of cells or surrogates of cells and methods for classifying cells and quantitative analysis of cell type, cell states and function. Cellular segmentation has been an intense focus in biomedical image analysis for decades and has evolved from largely *ad hoc* approaches to generalizable solutions (Meijering, 2012). The classification strategies for cell type (e.g., immune cell, epithelium, stromal, etc.) and state (e.g., injured, repairing, dividing) have developed rapidly (Meijering, 2012, 2020; Meijering et al., 2016). How different cell types organize into microenvironments or neighborhoods is import for our understanding of pathogenesis and biology. The identification and classification of these neighborhood or microenvironments is of significant interest to the bioimaging community (Allam et al., 2020; Stoltzfus et al., 2020; Solorzano et al., 2021). This mini review will cover the current state of quantitative analysis of tissues and cells in imaging data, with a discussion of segmentation, classification, and neighborhood analysis, specifically highlighting the application of machine learning, including recent advancements, challenges, and the tools available to the biomedical researchers.

## Segmentation

A cornucopia of segmentation approaches has been developed for specific experimental situations, tissue types or cell populations including clusters of cells, specific cell types, etc. (Meijering, 2012; Meijering et al., 2016). Often these approaches are built as pipelines in image processing software, enabling the sharing of segmentation methods (Berthold et al., 2007; de Chaumont et al., 2012; Schindelin et al., 2012; Bankhead et al., 2017; McQuin et al., 2018; Berg et al., 2019). A common approach is to first differentiate foreground, the cell, from background in a semantic segmentation step. Secondly, objects of interest in the image are isolated, or instance segmentation, by identifying and then splitting touching cells. Meijering outlined five fundamental methods for segmentation: intensity thresholding (Otsu, 1979), feature detection, morphologically based, deformable model fitting and region accumulation or splitting (Meijering, 2012). These methods are often combined sequentially. For instance, cell segmentation might include *semantic* segmentation of a foreground of all nuclei with pixel intensity, followed by a second *instance* segmentation for identifying an individual nucleus using a region accumulation approach like watershed (Beucher and Lantuejoul, 1979). A common limitation is the *ad hoc* nature of segmentation approaches: the applicability of a segmentation method may be limited by constraints in the datasets including differences in staining or imaging modality (fluorescence vs. histology staining), artifacts in image capture (out-of-focus light or uneven field illumination) or morphological differences (spherical epithelial vs. more cylindrical muscle nuclei). These constraints, and others, have limited the development of generalizable segmentation algorithms.

Cell segmentation with machine learning is well established-a popular approach is to perform semantic pixel segmentation with a Random Forest Classifier (Hall et al., 2009; McQuin et al., 2018; Berg et al., 2019). Segmentation with a Random Forest Classifier, as with all machine learning approaches, requires training data. In cell segmentation this is data that has been annotated to indicate which pixels in images are foreground, nuclei, vs. background. ilastik provides an intuitive and iterative solution for generating training data with a GUI that allows a user to: (1) highlight pixels to indicate nuclei vs. background-training data, (2) test classification and segmentation, (3) repeat and add or subtract highlighted pixels, to improve the classification and segmentation. This process is powerful but can become labor-intensive in different tissues where there may be a variety of nuclei (e.g., shape, texture, size, clustering, etc.) in smooth muscle, epithelium, endothelium, and immune cells in varying densities and distributions. Unfortunately, while high quality cell culture nuclei training datasets and tissue image datasets exist, 2D training data of nuclei in tissue is limited or fractured across multiple repositories (Ljosa et al., 2012; Williams et al., 2017; Ellenberg et al., 2018; Kume and Nishida, 2021). Furthermore, while 3D electron microscopy data is readily available, 3D fluorescence image or training datasets of nuclei is limited (Ljosa et al., 2012; Iudin et al., 2016; El-Achkar et al., 2021; Lake et al., 2021). The availability of training data is one of the most significant barriers to the application of machine learning to cell image segmentation (Ching et al., 2018). Fortunately, the number of venues to share imaging datasets should not limit the dissemination of training datasets as they are generated (Table 1, Datasets and Repositories).

**TABLE 1:** End user accessibility of tools supporting machine and/or deep learning for bioimage analysis.

User			Application				Name	Support or demonstrated		Description	Software type	URL	References
								Classical learning	Deep learning (DL)				
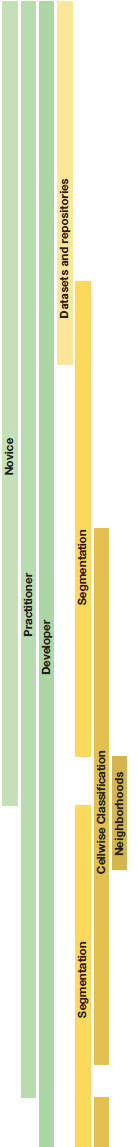							Image data resource (IDR)	Not determined	Yes, ex. Idr0042	Tissue and cell images with cell based training datasets	Repository	https://idr.openmicroscopy.org	Williams et al., 2017
							Broad bioimage benchmark collection	Yes	Yes	Cell images training datasets	Repository	https://bbbc.broadinstitute.org	Ljosa et al., 2012
							Cell image library	Not determined	CDeep3M	Mulitmodal cell images, linked to *CDeep3M* for testing	Repository	http://cellimagelibrary.org/pages/datasets	NA
							BioImageDbs	Yes	Yes	R package and repository for images	Bioconductor package	https://kumes.github.io/BioImageDbs/	Kume and Nishida, 2021
							EMPIAR	Yes, ex. EMPIAR-10069	Yes, ex. EMPIAR-10592	Electron microscopy images	Repository	https://www.ebi.ac.uk/empiar/	Iudin et al., 2016
							SciLifeLab	Not determined	Yes	Scientific data, images and figure	Repository	https://www.scilifelab.se/data/repository/	NA
							BioImage Archive	Yes	Yes	Archive of IDR and EMPIAR	Repository	https://www.ebi.ac.uk/bioimage-archive/	Ellenberg et al., 2018
							DeepCell Kiosk		Establishing a cellwise dataset	Tool for segmentation in the cloud	Web interface	deepcell.org	Moen et al., 2019
							Cellpose		Segmentation	Tool for segmentation in the cloud and python GUI	Web interface, application	https://github.com/MouseLand/cellpose	Stringer et al., 2021
							NucleAIzer		Transfer learning	Tool for segmentation in the cloud	Web interface	www.nucleaizer.org	Hollandi et al., 2020
							CDeep3M		Electron microscopy segmentation	Multiple trained networks for distinct structures in EM images	Web interface, model zoo	https://cdeep3m.crbs.ucsd.edu/	Haberl et al., 2018
							QuPath	Feature design for segmentation	Inference with StarDist	ML segmentation with GUI	Application, plugin	qupath.github.io	Bankhead et al., 2017
							DeepImageJ		Inference in ImageJ with BioImage.IO	Tool for inference on the desktop	Plugin	deepimagej.github.io/deepimagej/	Gómez-de-Mariscal et al., 2021
							Ilastik	Feature design for segmentation	Interfaces with BioImage.IO	Segmentation with GUI	Application, plugin	www.ilastik.org	Berg et al., 2019
							CellProfiler and CellAnalyst	Feature design for classification	Unet Segmentation	Pipeline Based image processing tool with ML and DL support	Application	cellprofiler.org	Dao et al., 2016; McQuin et al., 2018
							StarDist		Segmentation	Python and Java (ImageJ/FIJI) tool for segmentation	Plugin	https://github.com/stardist/stardist	Weigert et al., 2020
							HistomicsML2		Model for training and tools for inference	Framework for training and inference on imaging data	Web interface	https://histomicsml2.readthedocs.io/	Lee et al., 2021
							CSBDeep		Image restoration, segmentation	FIJI plugins and python for image restoration and segmentation	Python, plugin	https://csbdeep.bioimagecomputing.com/	Schmidt et al., 2018; Weigert et al., 2020
							CytoMAP	Feature design for neighborhoods		Cell classification and neighborhood analysis with GUI	Application	gitlab.com/gernerlab/cytomap/-/wikis/home	Stoltzfus et al., 2020
							Volumetric tissue exploration and analysis	Feature design for classification and segmentation		Cell segmentation, classification and neighborhood analysis with GUI	Plugin	https://vtea.wiki	Winfree et al., 2017
							modelzoo.co		Models for many datatypes	Open source and pretrained networks	Web repository	modelzoo.co	NA
							InstantDL		Segmentation and classification	Broadly applicable segmentation and classification framework	Python, CoLab	https://github.com/marrlab/InstantDL/	Waibel et al., 2021
							BioImage.IO		Models for specifically for bioimaging	DL networks for the bioimaging community	Web repository	bioimage.io	NA
							ZeroCostDL4Mic		Training and inference with BioImage.IO	Tool for training and inference in the cloud	Cloud based, CoLab	github.com/HenriquesLab/ZeroCostDL4Mic	von Chamier et al., 2021
							OpSeF		DL network training and inference	Python framework in Jupyter notebooks	Python	github.com/trasse/OpSeF-IV	Rasse et al., 2020
							Weka	Extensive library of classifiers and tools		ML frame work for Java, Plugin for ImageJ	API, application, plugin	www.cs.waikato.ac.nz/ml/weka/	Hall et al., 2009

Recently, three novel approaches were developed to address the dearth of segmentation training data for the variety of cell-types and imaging modalities. The first, and most direct approach has been the concerted effort of a number of groups including the Van Valen and Lundberg laboratories to establish “human-in-the-loop” pipelines and infrastructure of software and personnel, including collaborative crowd sourcing, to generate ground truth from imaging datasets (Sullivan et al., 2018; Moen et al., 2019; Bannon et al., 2021). A limitation of this approach is the requirement for on-going support for personnel; on-going support is critical to long term success. To ease the generation of high-quality training data with a “human-in-the-loop” approach, methods have also been established around segmentation refinement (Sullivan et al., 2018; Lutnick et al., 2019; Moen et al., 2019; Govind et al., 2021; Lee et al., 2021). An alternative to these brute-force approach has been to generate synthetic training data by combining “blob” models of cells with real images using generative adversarial networks (Dunn et al., 2019; Wu et al., 2021). Further, to leverage training data across imaging modalities NucleAIzer^1^ relies on style transfer with a generative-adversarial-network to generate synthetic data using prior training data from other modalities (fluorescence, histological stains, or immunohistochemistry). Thus, this approach can expand training data by mapping to a common modality, giving a nearly general solution to segmentation across 2D imaging modalities (Hollandi et al., 2020).

The on-going search for generalizable segmentation is an area of active research in deep learning and is critical to establishing rigorous and reproducible segmentation approaches. To this end, a pipeline that requires little to no tuning on multiple datasets and modalities was demonstrated recently (Waibel et al., 2021). In the interim, the field will continue to make progress with generalizable segmentation, existing approaches, networks, etc. can provide the foundation for novel segmentation solutions. For instance, deep learning approaches to address 2D and 3D cell segmentation are often based on existing networks (Haberl et al., 2018; Schmidt et al., 2018; Falk et al., 2019; Weigert et al., 2020; Minaee et al., 2021; Stringer et al., 2021), using training data augmentation (Moshkov et al., 2020), or transfer learning (Zhuang et al., 2021). Thus, until there is a generalizable solution, new deep learning segmentation approaches can be developed quickly by building on existing work with focused training datasets specific to tissue, cell-type and imaging modality.

## Classification

Using specific protein or structural markers is a common way to determine cell-types in cytometry approaches like flow and image cytometry. Image cytometry is complicated by defining which pixels are associated with which cells. While a nuclear stain can be used to identify the nucleus, membrane, and cytoplasmic markers may be inconsistent across cell-types, cell-states, and tissues. A common solution is to measure markers in pixels proximal to segmented nuclei. These pixels can be defined by using a limited cell-associated region-of-interest that wraps around an existing nuclear segmentation or by performing a tessellation with a Voronoi segmentation (Winfree et al., 2017; Goltsev et al., 2018; McQuin et al., 2018).

The mean-fluorescence intensity (or other intensity measure, mode, upper-quartile mean, etc.) of markers in cell-associated segmented regions is frequently used for classification. A common supervised approach is to perform a series of sequential selections or gates based on marker intensities like flow cytometry. This “gating strategy” can easily identify specific cell-types with a predefined cell-type hierarchy. Cell classification can be semi-automated with unsupervised or semi-supervised machine learning using classifiers and clustering approaches. Popular approaches include Bayesian and Random Forest classifiers and clustering with k-means or graph based community clustering like the Louvain algorithm (Hall et al., 2009; Dao et al., 2016; McQuin et al., 2018; Phillip et al., 2021; Solorzano et al., 2021). Importantly, analyzing highly multiplexed image datasets, more than twenty markers, with a supervised “gating” approach may prove intractable necessitating machine learning approaches (Levine et al., 2015; Goltsev et al., 2018; Neumann et al., 2021).

Deep learning has been broadly applied to classification of images (Gupta et al., 2019). One of the strengths of a deep learning classification approach, as with segmentation, is that it is possible to start with a pretrained network-potentially reducing training set sizes. For instance, in 2D image classification, a convolutional neural network (CNN) like ResNet-50 initially trained on natural images (e.g., animals, vehicles, plants, etc.) can be retrained with a new label structure and training data that might include, for instance, cell nuclei (Woloshuk et al., 2021). Some deep learning models can further simplify workflows, like regional-CNNs, performing both segmentation and classification (Caicedo et al., 2019).

One image dataset that presents an interesting challenge and unique opportunity in both segmentation and classification is multiplexed fluorescence *in situ* hybridization (FISH). These approaches can, through combinatorial labeling of fluorophores, generate images of nearly all putative transcripts (Coskun and Cai, 2016). Although a semantic and instance segmentation approach can be used to identify and classify cells using associated FISH probes (Littman et al., 2021), a recent pixelwise-segmentation free approach has been proposed. This approach organizes the detected FISH-probes into spatial clusters using graphs from which signatures of cells and cell-types are determined (Shah et al., 2016; Andersson et al., 2020; Partel and Wählby, 2021).

## Microenvironments as Neighborhoods

The classification of microenvironments in tissues informs our understanding of the role of specific cells and structures in an underlying biology. This has led to the development of neighborhood analysis strategies that involve the segmentation of groups of cells or structures which are then classified with machine learning using neighborhood features such as cell-type census and location (Stoltzfus et al., 2020; Solorzano et al., 2021; Winfree et al., 2021). This process mirrors the segmentation and classification of single cells by protein and RNA markers where the types of cells or the distributions of cell types in neighborhoods are the markers used to classify the neighborhoods. The segmentation strategies for defining neighborhoods usually rely on either regular sampling of a tissue or cell centric approaches (e.g., distance from a cell or the *k*-nearest neighborhoods) (Jackson et al., 2020; Stoltzfus et al., 2020; Lake et al., 2021; Winfree et al., 2021). The impact of neighborhood size and defining it variably and locally (e.g., microenvironments may be different near arterioles vs. microvasculature) are under explored avenues in the analysis of cellular microenvironments in bioimaging datasets. Importantly, further development of neighborhoods analyses is critical as it has demonstrated mechanistic insight in human disease when used with highly multiplexed chemical and fluorescence imaging (Jackson et al., 2020; Schürch et al., 2020; Stoltzfus et al., 2021).

## Technology and Tool Accessibility

Minimizing the exclusivity of segmentation and classification advancements with the development of user accessible tools, is critical to the democratization of image analysis. In the above discussions of both segmentation and classification, most researchers and developers paid careful attention to providing tools for use by biomedical scientists. Example tools include web interfaces, stand-alone applications, or plugins for open-source image processing software (Table 1). These tools provide access to users that are novices in image analysis, day-to-day practitioners and super-users or developers across the three fundamental tasks of cell segmentation, cell classification and neighborhood analysis (Table 1). Furthermore, deep learning networks are available through online repositories such as github.com and modelzoo.co. An exciting development is the recent set of publications that have defined one-stop-shops for deep learning models and accessible tools for using and training existing deep learning networks (Iudin et al., 2016; Berg et al., 2019; Rasse et al., 2020; Gómez-de-Mariscal et al., 2021; von Chamier et al., 2021, p. 4). This includes the integration of segmentation tools with online repositories of trained deep learning networks that can be easily downloaded and tested on cells and modalities of interest. With this added accessibility, there is a risk of misuse and possible abuse. However, the ease of reproducibility may outweigh this risk.

## Conclusion

The bioimaging community has recognized for decades that image data is more than a picture. Mining imaging data collected in the biomedical sciences has blossomed in the past 20 years, pushed by advancements in multiplexed tissue labeling, image capture technologies, computational capacity, and machine learning. It will be exciting to see the next developments in image analysis with machine learning approaches. Perhaps we will witness: (1) a fully generalizable multidimensional cell segmentation approach; (2) novel approaches to cell-segmentation independent of pixelwise classification (as with some FISH data), or (3) new models of neighborhoods to characterize cellular microenvironments and niches. Furthermore, with web-based repositories to share datasets and tools that are suitable for all levels of expertise, these and other developments will be accessible to both experts, practitioners, *and* researchers new to imaging and image analysis. The broad accessibility of image data and tools could facilitate the adoption of common and rigorous processes for meaningful biological insight from image datasets across fields of study for so much more than a picture.

## Author Contributions

SW conceived and outlined the manuscript.

## Conflict of Interest

The author declares that the research was conducted in the absence of any commercial or financial relationships that could be construed as a potential conflict of interest.

## Publisher’s Note

All claims expressed in this article are solely those of the authors and do not necessarily represent those of their affiliated organizations, or those of the publisher, the editors and the reviewers. Any product that may be evaluated in this article, or claim that may be made by its manufacturer, is not guaranteed or endorsed by the publisher.

## Footnotes

1
https://www.nucleaizer.org/

## Abbreviations

ML: machine learning
DL: deep learning
GUI: graphical user interface
API: application programming interface.
